# Rapid screening for depression – validation of the Brief Case-Find for Depression (BCD) in medical oncology and palliative care patients

**DOI:** 10.1038/sj.bjc.6602057

**Published:** 2004-08-10

**Authors:** M Jefford, L Mileshkin, K Richards, J Thomson, J P Matthews, J Zalcberg, R Jennens, S-A McLachlan, S Wein, Y Antill, D M Clarke

**Affiliations:** 1 Department of Haematology and Medical Oncology, Peter MacCallum Cancer Centre, Locked Bag 1, A'Beckett Street, Victoria 8006, Australia; 2 Statistical Centre, Peter MacCallum Cancer Centre, Melbourne, Victoria, Australia; 3 Department of Psychological Medicine, Monash Medical Centre, Melbourne, Victoria, Australia

**Keywords:** depression, mass screening, medical oncology, palliative care

## Abstract

Depression in oncology patients is under-recognised and associated with poor outcomes. Screening can increase case recognition. The Brief Case-Find for Depression (BCD) is a four-question, interviewer-administered instrument that has been previously validated in a general medical setting. The primary aim of this study was to validate the BCD in a medical oncology/palliative care setting, primarily by comparing its association with physical illness measures and with the Primary Care Evaluation of Mental Disorders (PRIME-MD), the Beck Depression Inventory (BDI) and the Hospital Anxiety and Depression Scale (HADS). Eligible adult oncology patients gave informed consent and completed the above measures and a pain scale. Agreement between the BCD and other instruments was assessed. Construct validity was determined by comparing depressed/nondepressed patients with respect to performance status, symptoms, pain score and functional impairment. A total of 100 patients had a median age of 58 (range 21–90) and ECOG performance status (PS) 2 (0–4). In all, 60% had metastatic disease. The therapeutic goal was curative/adjuvant in 39% and palliative in 61%. Prevalence of depression according to the various measures was: BCD 34%, PRIME-MD 12%, BDI 19% and HADS 14%. In total, 45% of patients responded positively to a single-item screening question. The BCD showed fair agreement with the PRIME-MD (kappa=0.21), moderate agreement with the BDI (kappa=0.43) and fair agreement with the HADS (kappa=0.27). Against the PRIME-MD diagnosis of depression, the BCD had greater sensitivity, but lesser specificity and overall agreement, compared with the BDI and depression scale of the HADS. Patients with probable depression (according to BCD) had inferior PS (*P*=0.0064), increased pain (*P*=0.045) and greater impairment of functioning (PRIME-MD: *P*=0.0003). There was no association with gender, age, disease status or therapeutic goal. Depression is common in oncology patients. The BCD is a quick, easy-to-administer screen for depression, which has reasonable psychometric properties in this population.

Depression is common in medical oncology and palliative care patients. The reported incidence of major depression at any point during the cancer journey varies greatly because of methodological differences and inconsistencies, though it is often reported to be around 25% (Bukberg *et al*, 1984; Lansky *et al*, 1985; Kathol *et al*, 1990; Massie and Holland, 1990; Portenoy *et al*, 1994). Depression appears to be more common in patients with pain, advanced illness and higher levels of disability (Bukberg *et al*, 1984; Lansky *et al*, 1985; Katon and Sullivan, 1990; Massie and Holland, 1990; Massie and Holland, 1992; Spiegel *et al*, 1994).

There is considerable evidence that physicians and nursing staff under-recognise depression in oncology patients (Hardman *et al*, 1989; Ford *et al*, 1994; Newell *et al*, 1998; Passik *et al*, 1998; McDonald *et al*, 1999). Underdiagnosis of depression may result in poor patient outcomes as well as impact upon others, such as family and friends. Depression may be associated with a reduced ability to enjoy life and to participate in social interactions, as well as poor compliance with medical treatment, inferior pain control, increased hospitalisation and an increased risk of suicide (Breitbart, 1987; Spiegel, 1996; Block, 2000; DiMatteo *et al*, 2000).

Treatment for depression in the oncology and palliative care settings has been shown to be effective. Optimal management combines supportive psychotherapy, cognitive–behavioural techniques and antidepressant medications (Massie and Holland, 1990; Spiegel, 1996; Block, 2000; Gill and Hatcher, 2003).

Diagnostic criteria for a major depressive episode are defined within the Diagnostic and Statistical Manual of Mental Disorders of the American Psychiatric Association (DSM-IV, 1994). The diagnosis requires a constellation of symptoms, present for at least 2 weeks and representing a change from previous functioning. At least one of the symptoms must be either depressed mood or loss of interest or pleasure (anhedonia). Some patients may not satisfy the DSM-IV diagnostic criteria for a major depressive episode but may still suffer from significant psychological morbidity. A range of additional disorders including adjustment disorder with depressed mood can also be recognised and may benefit from treatment (Hotopf *et al*, 2002).

Depression may be distinguished from normal sadness and anticipatory grief on the basis of the nature and severity of symptoms, their duration and intensity and their impact upon functioning. The clinical psychiatric interview is often considered the gold standard for the diagnosis of depression. However, such consultations are time-consuming, not always available and may be inconvenient for patients already requiring multiple hospital or clinic visits. Therefore, in an effort to improve the recognition of depression, a number of screening instruments have been developed. Few such instruments have been studied in the oncology setting and none have been implemented into routine clinical care.

The Primary Care Evaluation of Mental Disorders (PRIME-MD) Patient Health Questionnaire is a validated tool for the diagnosis of mental disorders. The patient-completed questionnaire is a simple, though structured way to establish a diagnosis of major depressive syndrome and panic syndrome, according to DSM-IV symptom criteria, though excluding the duration criteria (Spitzer *et al*, 1999). In addition to making a criteria-based diagnosis of a depressive disorder, the PRIME-MD is also a reliable and valid measure of depression severity (Kroenke *et al*, 2001). The Beck Depression Inventory (BDI) (Beck *et al*, 1961; Richter *et al*, 1998) and the Hospital Anxiety and Depression Scale (HADS) (Zigmond and Snaith, 1983) are other commonly used, patient-completed screening instruments. However, there have been concerns expressed that these instruments may not be ideal screening tools within the oncology setting (Urch *et al*, 1998; Love *et al*, 2002), with the PRIME-MD appearing to have operating characteristics superior to HADS (Lowe *et al*, 2004).

The Brief Case-Find for Depression (BCD – Figure 1

**Figure 1:**
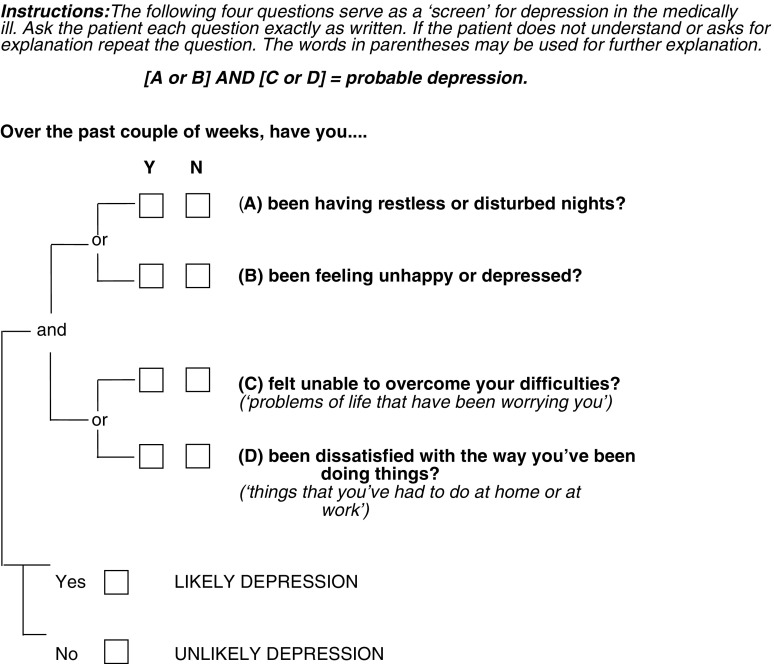
The Brief Case-Find for Depression (BCD).

) (Clarke *et al*, 1994) is a further simple screening tool that has been used within general medical and geriatric populations to identify patients with depression. It is a clinician-completed four-question instrument intended to be administered during the course of a normal clinical interview. As the clinician completes the instrument in around 1 min, it is particularly suitable for patients who are frail or exhausted. Component items were derived from the General Health Questionnaire (GHQ) (Goldberg, 1972), facilitated by a computer-assisted technique that sought to find the best items to identify both major depression and minor depression (adjustment disorders) (Clarke *et al*, 1994). The BCD is acceptable to patients and easy to administer and score. In a validation study, performed in the setting of a general teaching hospital, the BCD performed better than the standard GHQ, HADS and BDI (Clarke *et al*, 1994). The utility of the BCD in patients with cancer has not been formally assessed.

The primary aim of this trial, therefore, was to validate the BCD in a medical oncology and palliative care setting, firstly by assessing for agreement with factors known to associate with depression, such as pain and poor performance status, and, secondly, by comparing the performance of the BCD with the PRIME-MD and also with the BDI and HADS. A further aim was to determine the prevalence of ‘cases’ of likely depression (as determined by the BCD, PRIME-MD, BDI and HADS) in this patient care setting.

Chochinov *et al* (1997) have suggested that a single-item screen, essentially asking ‘Are you depressed?’ is an accurate screen for depression. We therefore also assessed the performance of question 2 of the BCD (‘Over the past couple of weeks, have you been feeling unhappy or depressed?’) operating as a single-item screen.

## MATERIALS AND METHOD

### Eligibility

To be eligible, patients were required to have a histological or cytological diagnosis of cancer, be aged 18 or older, be able to independently complete the English version of the PRIME-MD, BDI, HADS and McGill Melzack Pain Questionnaire (Walsh and Bowman, 1982) and were required to provide written informed consent. Exclusion criteria included dementia or other psychiatric illness and the presence of a cognitive disorder that might preclude accurate completion of the patient-completed measures. Patients considered by their physician to be too debilitated to complete the study questionnaires were also excluded. The study was approved by the ethics committee of the Peter MacCallum Cancer Centre.

### Study design

A target sample size of 100 eligible patients was chosen for pragmatic reasons. After each patient had provided informed consent, the investigating doctor completed the BCD. The patient then completed, in order, the self-report version of the PRIME-MD, the BDI and HADS. Patients also completed the Present Pain Intensity component of the McGill Melzack Pain Questionnaire. The doctor administering the BCD scored the BCD and the PRIME-MD immediately after their completion. The patient's treating doctor was notified of any patient recognised as being probably depressed based on either the BCD or the PRIME-MD, and was responsible for managing the patient in accordance with treatment recommendations specified within the protocol regarding counselling and the use of antidepressant medications.

### Statistical analysis

#### Quality control

Data were entered into a project-specific Microsoft Access (Microsoft Corporation, Redmond, WA, USA) database and were independently verified using a double-data-entry procedure.

#### Scoring of the instruments

Brief Case-Find for Depression: Patients were scored as a probable case of depression if they answered yes to at least one of questions A and B and to at least one of questions C and D (see Figure 1).

PRIME-MD: Patients were scored as having a ‘major depressive syndrome’ on the PRIME-MD if they answered at least ‘more than half the days’ to questions 1a OR 1b AND to five or more of questions 1a–i, with the exception that 1i was scored positively if there was any positive response. Patients were scored as having an ‘other depressive syndrome’ if they answered at least ‘more than half the days’ to questions 1a OR 1b AND to two, three or four of questions 1a–i, with the exception that 1i was scored positively if there was any positive response. Patients were scored as having a panic syndrome if they answered ‘yes’ to all of questions 2a–e.

Beck Depression Inventory: Each of the 21 questions was scored on a four-point scale (0–3) and aggregated. Cutoff scores were those commonly used (Richter *et al*, 1998), specifically: <10 – none or minimal depression, 10–18 – mild to moderate depression, 19–29 – moderate to severe depression and 30–63 – severe depression.

Hospital Anxiety and Depression Scale: The seven depression questions were scored on a four-point scale (0–3) and aggregated. Cutoff scores used were those commonly used (Herrmann, 1997), specifically: <7 – non-case, 8–10 – doubtful case, ⩾11 – definite case. The seven anxiety items were scored and aggregated in the same way.

#### Comparison of screening tools

The PRIME-MD was used as a surrogate standard for the diagnosis of a major depressive episode, as this instrument essentially represents a checklist for the DSM-IV diagnostic criteria for major depressive episode. Kappa values were calculated to determine the correspondence between the BCD and PRIME-MD and with BDI and HADS. The strength of agreement has been interpreted as given by Landis and Koch (1977). The percentage agreement between the PRIME-MD and each of the screening tools was also calculated, without correcting for chance, calculated simply as the percentage of cases with the same classification on both tools. Similarly, the sensitivity and specificity of the screening tools were calculated relative to the PRIME-MD. Sensitivity was calculated as the percentage of cases on the PRIME-MD that were positive on the screening instruments and specificity was calculated as the percentage of negative cases on the PRIME-MD that were negative on the screening instruments. The positive predictive value was calculated as the percentage of positive cases on the screening instruments that were cases on the PRIME-MD. The negative predictive value was calculated as the percentage of negative cases on the screening instruments that were truly negative on the PRIME-MD.

#### Pre-specified hypotheses

The study tested the following hypotheses, established prospectively and stated in the protocol.

1. Depressed patients, compared to nondepressed patients, as determined by the BCD, will have:
  - Higher scores on the global self-assessment of the degree of difficulty in functioning due to problems on the PRIME-MD
  - Poorer ECOG performance status
  - Higher pain scores
  - More advanced/symptomatic disease.
2. There will be at least moderate agreement (kappa>0.4) between:
  - Cases as determined by the BCD and PRIME-MD diagnosis of major depression
  - BCD and BDI
  - BCD and the HADS depression subscale.
3. There will be only fair agreement (kappa>0.2) between:
  - Positive cases on the BCD and positive cases on the panic scale of the PRIME-MD (patients coded as having a panic syndrome)
  - Positive cases on the anxiety scale of the HADS.

#### Methods of analysis

Confidence intervals (CIs, 95%) have been reported for the major summary statistics. For sensitivity and specificity estimates and other binary outcomes, they have been based on the Blyth–Still–Casella method (StatXact 5.0.3. CYTEL Software Corporation, 2001). Comparisons of depressed and nondepressed patients with respect to binary outcome variables have been made using Fisher's exact test. The Cochran–Armitage test for trend was used for the comparison of ordinal data and the Wilcoxon–Mann–Whitney test was used for comparison of ages. One-sided tests of significance were used where positive associations were hypothesised in the protocol, otherwise two-sided tests were used. No adjustment has been made for multiple comparisons.

All statistical analyses were carried out using StatXact (StatXact 5.0.3. CYTEL Software Corporation, 2001) or SPSS (SPSS for Windows 11.0.1 SPSS Inc, 2001) statistical software.

## RESULTS

### Patient characteristics and association with probable depression on BCD

A total of 102 patients were registered on the trial between October 2001 and December 2002. One patient was found to be ineligible, being aged 16 at the time of registration, and was thus excluded from the trial. A second patient was eligible for the trial and was evaluated with respect to the BCD. However, the patient felt too unwell to complete the remaining questionnaires and was withdrawn from the study.

Patient characteristics are given in Table 1

**Table 1:** Patient characteristics and association with probable depression on BCD

	**Probable depression on BCD**			
	**No**	**Yes**	**Total**	***P*-value**
*Sex*				
Male	34 (63%)	20 (37%)	54	0.53^a^
Female	32 (70%)	14 (30%)	46	
				
*Age group*				
⩽30	3 (43%)	4 (57%)	7	
30–39	5 (50%)	5 (50%)	10	
40–49	9 (69%)	4 (31%)	13	
50–59	16 (62%)	10 (38%)	26	0.11^b^
60–69	22 (76%)	7 (24%)	29	
70–79	9 (75%)	3 (25%)	12	
⩾80	1 (50%)	1 (50%)	2	
				
*Living arrangements*				
With others	56 (64%)	31 (36%)	87	0.53^a^
Alone	10 (77%)	3 (23%)	13	
				
*ECOG performance status*				
0	15 (94%)	1 (6%)	16	
1	26 (67%)	13 (33%)	39	
2	16 (57%)	12 (43%)	28	0.0064^c^
3	9 (56%)	7 (44%)	16	
4	0 (0%)	1 (100%)	1	
				
*Type of cancer*				
Gastrointestinal	18 (78%)	5 (22%)	23	
Breast	14 (67%)	7 (33%)	21	
Head and neck	7 (70%)	3 (30%)	10	
Sarcoma	6 (60%)	4 (40%)	10	
Skin	5 (71%)	2 (29%)	7	0.31^d^
Gynaecological	3 (50%)	3 (50%)	6	
Lung	2 (33%)	4 (67%)	6	
Haematological	4 (100%)	0 (0%)	4	
Other^e^	7 (54%)	6 (46%)	13	
				
*Disease status*				
No evidence of disease	12 (75%)	4 (25%)	16	0.88^d^
Localised disease, asymptomatic	2 (50%)	2 (50%)	4	
Localised disease, symptomatic	13 (65%)	7 (35%)	20	
Metastatic disease, asymptomatic	7 (70%)	3 (30%)	10	
Metastatic disease, symptomatic	32 (64%)	18 (36%)	50	
No evidence of disease	12 (75%)	4 (25%)	16	
Localised disease	15 (63%)	9 (38%)	24	0.34^c^
Metastatic disease	39 (65%)	21 (35%)	60	
				
*Symptoms*				
Absent	21 (70%)	9 (30%)	30	0.38^f^
Present	45 (64%)	25 (36%)	70	
				
*Pain*				
No pain	27 (77%)	8 (23%)	35	
Mild pain	14 (70%)	6 (30%)	20	
Discomforting pain	15 (60%)	10 (40%)	25	0.045^c^
Distressing pain	6 (40%)	9 (60%)	15	
Horrible pain	3 (75%)	1 (25%)	4	
Excruciating pain	1 (100%)	0 (0%)	1	
				
*Goals of therapy*				
Curative/adjuvant	26 (67%)	13 (33%)	39	1.00^a^
Palliative	40 (66%)	21 (34%)	61	

a Fisher's exact test (two-sided).

b Wilcoxon–Mann–Whitney test using actual ages (two-sided).

c Cochran–Armitage test for trend (one-sided).

d Pearson *χ*^2^ test (two-sided).

e Other cancers were as follows: testicular seminoma, three; metastatic carcinoma of unknown primary, two; soft tissue neuroendocrine tumour, one; renal cell carcinoma, one; prostate, one; phaeochromocytoma, one; peripheral neuroectodermal tumour, one; medullary thyroid carcinoma, one; fibrous histiocytoma, one; carcinoid tumour, one.

f Fisher's exact test (one-sided).

. The median age was 58 years (range 21–90). As anticipated, there was a positive association between depression as identified on the BCD and ECOG performance status (*P*=0.0064) and higher pain scores (*P*=0.045). There was no association with the extent of disease (none, localised or metastatic) (*P*=0.34) or the presence of symptoms (*P*=0.38).

### Prevalence of depression in the study population

Overall, 34% of patients were scored as likely cases of depression on the BCD, compared with 12% on the PRIME-MD, 19% on the BDI and 14% on the HADS (Table 2

**Table 2:** Prevalence of depression in the study population

	**Prevalence (%)**	**95% CI (%)**
BCD (likely depression)	34	25–44
PRIME-MD (major depressive syndrome)	12	7–19
PRIME-MD (major or other depression)	21	13–30
BDI (moderate or severe depression – BDI ⩾19)	19	12–28
BDI (mild, moderate or severe – BDI ⩾10)	43	33–53
HADS (definite case, HADS ⩾11)	14	8–22
HADS (doubtful or definite case, HADS ⩾8)	19	12–28

). Using less stringent cutoff scores for caseness, the prevalence of possible depression was considerably higher on the latter instruments (Table 2).

### Association between the BCD and other screening tools

The association between the BCD and other instruments is shown in Table 3

**Table 3:** Association between BCD and other screening/diagnostic tools

	**Probable depression on BCD**			
	***N* (row percent)**			
	**No**	**Yes**	**Total**	***P*-value**
*Major depression on PRIME-MD*				
No	62 (70%)	26 (30%)	88	
Yes	4 (33%)	8 (67%)	12	0.015^a^
				
*Other depression on PRIME-MD*				
No	63 (69%)	28 (31%)	91	
Yes	3 (33%)	6 (67%)	9	0.039^a^
				
*Major or other depression on PRIME-MD*				
No	59 (75%)	20 (25%)	79	
Yes	7 (33%)	14 (67%)	21	0.0006^a^
				
*Degree of difficulty in functioning due to problems identified on PRIME-MD*^b^				
Not difficult at all	39 (83%)	8 (17%)	47	
Somewhat difficult	19 (49%)	20 (51%)	39	
Very difficult	2 (33%)	4 (67%)	6	
Extremely difficult	1 (33%)	2 (67%)	3	0.0003^c^
				
*BDI depression score*				
Mean (s.d.)	7.7 (5.3)	18.1 (9.7)	11.2 (8.6)	
Median (range)	6 (0–21)	17.5 (4–40)	8 (0–40)	<0.0001^d^
				
*BDI depression group*				
None or minimal	49 (86%)	8 (14%)	57	
Mild	13 (54%)	11 (46%)	24	
Moderate	4 (27%)	11 (73%)	15	
Severe	0 (0%)	4 (100%)	4	<0.0001^c^
				
*HADS depression score*				
Mean (s.d.)	3.2 (3.2)	7.4 (4.6)	4.6 (4.2)	
Median (range)	2 (0–14)	6 (2–19)	3 (0–19)	<0.0001^d^
				
*HADS depression group*				
Non-case	60 (74%)	21 (26%)	81	
Doubtful case	2 (40%)	3 (60%)	5	
Definite case	4 (29%)	10 (71%)	14	0.0006^c^

a Fisher's exact test (one-sided).

b Excludes three patients who did not record any problems on PRIME-MD and two patients who recorded problems but did not answer question 3.

c Wilcoxon–Mann–Whitney test (one-sided).

d Cochran–Armitage test for trend (one-sided).

. Agreement with the BCD was fair for the PRIME-MD (kappa=0.21), moderate for the BDI (kappa=0.43) and fair for the depression subscale of the HADS (kappa=0.27). There was only slight agreement between positive cases on the BCD and positive cases on the anxiety scale of the HADS (kappa=0.19) and poor agreement between positive cases on the BCD and positive cases on the panic scale of the PRIME-MD (kappa=−0.02).

Patients with probable depression on BCD had significantly higher BDI scores and HADS depression scores than patients without probable depression on BCD (*P*<0.0001 for all comparisons).

As hypothesised, depressed patients according to the BCD, compared to nondepressed patients, had higher scores on the PRIME-MD global self-assessment of the degree of difficulty in functioning due to problems (*P*=0.0003).

### Association of BCD, BDI and HADS with PRIME-MD

Agreement of the BCD, BDI and HADS with the PRIME-MD is illustrated in Table 4

**Table 4:** Comparison of screening tools with the PRIME-MD major depression

		**BDI**	**HADS**
	**BCD**	**(score ⩾19)**	**(score ⩾11)**
Base rate of screening positive	34%	19%	14%
Kappa	0.34	0.44	0.49
% agreement	73%	82%	85%
Sensitivity	67%	52%	48%
Specificity	75%	90%	95%
Positive predictive value	41%	58%	71%
Negative predictive value	89%	88%	87%

. Both BDI and HADS had a greater overall agreement with the PRIME-MD than the BCD. The BCD had superior sensitivity.

### Single item

We examined the responses to question 2 of the BCD, ‘Over the past couple of weeks, have you been feeling unhappy or depressed?’ In all, 45% of patients answered ‘yes’ to this question. However, 24% of cases who were scored as having probable depression on the BCD answered ‘no’ to this item. Compared with the BCD as a whole, the single item had lower concordance with the PRIME-MD – either for ‘major depressive syndrome’ or for ‘other depressive syndrome’.

## DISCUSSION

We aimed to validate the use of the BCD in a medical oncology and palliative care setting. The BCD is a simple instrument that can be administered by the treating professional in around 1 min as part of the routine clinical interview, with results available immediately. We anticipated that, if the BCD demonstrated reasonable psychometric properties, it could be introduced into standard clinical practice.

We found fair agreement between the BCD and PRIME-MD, moderate agreement between positive cases on the BCD and BDI and fair agreement between the BCD and HADS. This variation in levels of agreement suggests that the BCD, the HADS and the PRIME-MD are not measuring exactly the same construct. The prevalence rate of depression was also different for the different instruments, suggesting that each may have different thresholds of severity for caseness. The BCD is a broad screen (with a base rate of screening positive of 34%) and therefore tends to be over-inclusive, but has good sensitivity and negative predictive power, though poorer specificity. As it is a broad screen, it is not surprising that agreement is greater with the BDI and lesser with the PRIME-MD (which identifies major depression) and the HADS (which focuses on anhedonic depression).

As expected, patients with probable depression on BCD had significantly higher BDI scores, HADS depression scores and HADS anxiety scores compared with patients without probable depression. Additionally, depressed patients according to the BCD, compared to nondepressed patients, had significantly higher scores on the PRIME-MD global self-assessment of the degree of difficulty in functioning due to problems. These results suggest good convergent validity of the BCD.

As anticipated, we found only slight agreement between positive cases on the BCD and positive cases on the anxiety scale of the HADS, and poor agreement between cases on the BCD and positive cases on the panic scale of the PRIME-MD. This indicates some distinction between depression and anxiety states and provides support for the discriminant validity of the BCD.

As others have noted, depression was more frequently diagnosed in patients with increased pain (Massie and Holland, 1990, 1992; Spiegel *et al*, 1994). We also confirmed the reported association between probable depression and impaired functional capacity (Bukberg *et al*, 1984; Lansky *et al*, 1985; Katon and Sullivan, 1990; Portenoy *et al*, 1994; Weitzner *et al*, 1997). This was seen in the association with ECOG performance status and with the PRIME-MD item ‘degree of difficulty in functioning due to problems’.

The sensitivity of the BCD was higher than the other instruments. We anticipate that a positive result on a screening instrument should prompt a more thorough psychiatric assessment. Due to the superior sensitivity, the prevalence of likely depression, as measured by the BCD, was higher compared with the other instruments. Although screening instruments will yield some false-positive results, it is preferable that a screening instrument be over-inclusive, rather than miss potential cases. Of the 100 patients screened, 26 cases of probable depression on the BCD were not diagnosed as having a major depressive syndrome according to the PRIME-MD. This might be expected as PRIME-MD cases approximate the diagnostic criteria for major depression, whereas the BCD was designed to recognise both major and minor depression. The BCD did not detect four cases of major depressive syndrome identified on the PRIME-MD. It is uncertain whether these cases represent true false negatives. Certainly all patients with probable depression should be followed with an appropriate diagnostic interview.

Concerning the effectiveness of a single-item screen, the study by Chochinov *et al* (1997) has been critiqued by Swanwick and Wrigley (1998), who have suggested that many older patients may fail to complain of low mood. A recent study by Lloyd-Williams *et al* (2003) also found that a similar single item had relatively low sensitivity (55%) and specificity (74%) when compared to a semistructured clinical psychiatric interview. It is possible that some depressed people acknowledge anhedonia but do not acknowledge feeling depressed. In this study, around a quarter of all cases answered ‘no’ to the relevant single item. This is clearly a limitation of a single-item screen.

In this study, we did not assess the impact of case finding on the subsequent management of patients. The importance of detecting possible cases of depression is that this should prompt further assessment and, if necessary, management of cases of likely depression. Our study aimed, primarily, to determine the validity of the BCD. We are now investigating the feasibility of routine implementation of the BCD, initially for hospitalised patients. Our approach incorporates education and training for resident medical officers regarding depression in general, administration of the BCD, responding appropriately to emotional cues/distress and facilitation of referral to psycho-oncology services. The implementation pilot will assess the impact of the routine administration of the BCD on staff practice/satisfaction/workload (resident medical staff, psycho-oncology and ward staff) and on patient outcomes (referrals, final psychiatric diagnosis, treatments and response to treatments).

## CONCLUSIONS

Depression is common in a medical oncology and palliative care setting. We found that the BCD is a simple screen, which is easy to administer and has good psychometric properties – good sensitivity and negative predictive power. We confirmed previously noted associations between depression and both increased pain and impaired functional capacity. The BCD also showed concordance with other depression screening tools. The BCD proved simple for patients and professionals to use. Further work will aim at studying the impact of routine implementation of the BCD and will assess the impact of the BCD upon physician practices and upon patient outcomes.
